# Structural Basis of DNMT1 and DNMT3A-Mediated DNA Methylation

**DOI:** 10.3390/genes9120620

**Published:** 2018-12-11

**Authors:** Wendan Ren, Linfeng Gao, Jikui Song

**Affiliations:** 1 Department of Biochemistry, University of California, Riverside, CA 92521, USA; wendan@ucr.edu; 2 Environmental Toxicology Program, University of California, Riverside, CA 92521, USA; lgao010@ucr.edu

**Keywords:** DNMT1, DNMT3A, DNA methyltransferase, maintenance DNA methylation, de novo DNA methylation, allosteric regulation, autoinhibition

## Abstract

DNA methylation, one of the major epigenetic mechanisms, plays critical roles in regulating gene expression, genomic stability and cell lineage commitment. The establishment and maintenance of DNA methylation in mammals is achieved by two groups of DNA methyltransferases (DNMTs): DNMT3A and DNMT3B, which are responsible for installing DNA methylation patterns during gametogenesis and early embryogenesis, and DNMT1, which is essential for propagating DNA methylation patterns during replication. Both groups of DNMTs are multi-domain proteins, containing a large N-terminal regulatory region in addition to the C-terminal methyltransferase domain. Recent structure-function investigations of the individual domains or large fragments of DNMT1 and DNMT3A have revealed the molecular basis for their substrate recognition and specificity, intramolecular domain-domain interactions, as well as their crosstalk with other epigenetic mechanisms. These studies highlight a multifaceted regulation for both DNMT1 and DNMT3A/3B, which is essential for the precise establishment and maintenance of lineage-specific DNA methylation patterns in cells. This review summarizes current understanding of the structure and mechanism of DNMT1 and DNMT3A-mediated DNA methylation, with emphasis on the functional cooperation between the methyltransferase and regulatory domains.

## 1. Introduction

DNA methylation represents one of the major epigenetic mechanisms that critically influence gene expression and cell fate commitment [1,2,3,4,5,6]. In mammals, DNA methylation is essential for the silencing of retrotransposons [7,8,9], genomic imprinting [10,11] and X-chromosome inactivation [12,13]. Mammalian DNA methylation predominantly occurs at the C-5 position of cytosine within the CpG dinucleotide context, accounting for ~70–80% of CpG sites throughout the genome [14]. The establishment of DNA methylation is achieved by the closely related DNA methyltransferases 3A (DNMT3A) and 3B (DNMT3B), designated as de novo DNA methyltransferases, during germ cell development and early embryogenesis [15,16]. Subsequently, clonal transmission of specific DNA methylation patterns is mainly mediated by DNA methyltransferase 1 (DNMT1), designated as maintenance DNA methyltransferase, in a replication-dependent manner [17,18]. However, the classification of DNMT3A/3B as de novo methyltransferases and DNMT1 as maintenance DNA methyltransferase appears to be an oversimplification, as increasing evidence has revealed an important role of DNMT3A and DNMT3B in DNA methylation maintenance [19,20], while other studies have pointed to the de novo methylation activity of DNMT1 in specific loci [21,22]. A detailed understanding of the structure and regulation of DNMT1 and DNMT3A/3B is essential for elucidating their roles in DNA methylation maintenance and establishment in cells.

Both DNMT1 and DNMT3A/3B belong to the class I methyltransferase family [23], featured by a conserved catalytic core termed Rossmann fold, which consists of a mixed seven-stranded β-sheet flanked by three α-helices on either side [24]. These enzymes catalyze the methylation reaction in an S-adenosyl-L-methionine (AdoMet)-dependent manner, with the catalytic core harboring essential motifs for enzymatic catalysis and cofactor binding. In addition, a subdomain, termed target recognition domain (TRD), is inserted between the central β-sheet and the last α-helix of the catalytic core [24]. The TRD bears no sequence similarity between DNMT1 and DNMT3s; instead, it participates in DNA binding to ensure substrate specificity of each enzyme.

**Figure 1:**
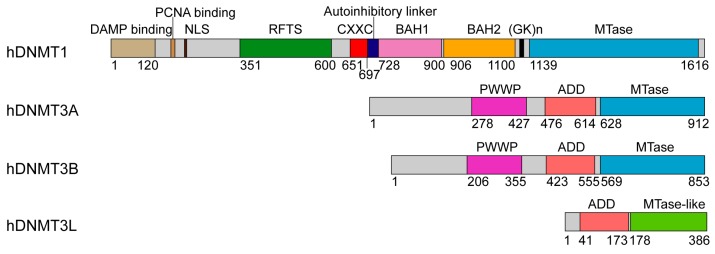
Domain architectures of human DNA methyltransferases: DNMT1, DNMT3A and DNMT3B, and regulator DNMT3L, with individual domains marked by residue numbers.

To ensure proper programming of DNA methylation patterns in cell linage commitment, the functions of DNMTs are subject to a stringent regulation during development [25,26]. Unlike their bacterial counterparts that contain only the methyltransferase (MTase) domain, both DNMT1 and DNMT3s are multi-domain proteins, containing a large regulatory region in addition to the C-terminal MTase domain (Figure 1) [18,27]. Recent studies have generated a large body of structural and functional information on both groups of enzymes, including the molecular basis underlying their enzyme-substrate recognition, and the regulatory roles of their N-terminal segments in the substrate specificity, enzymatic activity as well as genomic targeting. This review provides an overview on the recent progress in structural and mechanistic understanding of DNMT1 and DNMT3A, with an emphasis on how the regulatory and MTase domains of each enzyme cooperate in maintenance and de novo DNA methylation, respectively. 

## 2. Structure and Mechanism of DNMT1

DNMT1 is comprised of ~1600 amino acids, with an N-terminal regulatory region covering two thirds of the sequence, a highly conserved (GK)n repeat and a C-terminal MTase domain (Figure 1). The regulatory region starts with a ~300 amino acid-long N-terminal domain (NTD) harboring a variety of protein and/or DNA interaction sites, followed by a replication foci-targeting sequence (RFTS) domain, a CXXC zinc finger domain and a pair of bromo-adjacent-homology (BAH) domains (Figure 1). The function of DNMT1 in replication-dependent DNA methylation maintenance is supported by its localization in replication foci during the S phase, and in vitro a 3–40 fold enzymatic preference for hemimethylated CpG sites [18,28], an epigenetic mark enriched at the replication foci [29]. How the regulatory domains of DNMT1 are coordinated in attaining its enzymatic and spatiotemporal regulations remains a long-lasting topic of interest. Nevertheless, recent structure-function studies of various DNMT1 fragments under different DNA binding states [30,31,32,33] have started to illuminate how different domains of this enzyme orchestrate its activity in maintenance DNA methylation.

### 2.1. Enzyme-Substrate Interaction of DNMT1

**Figure 2:**
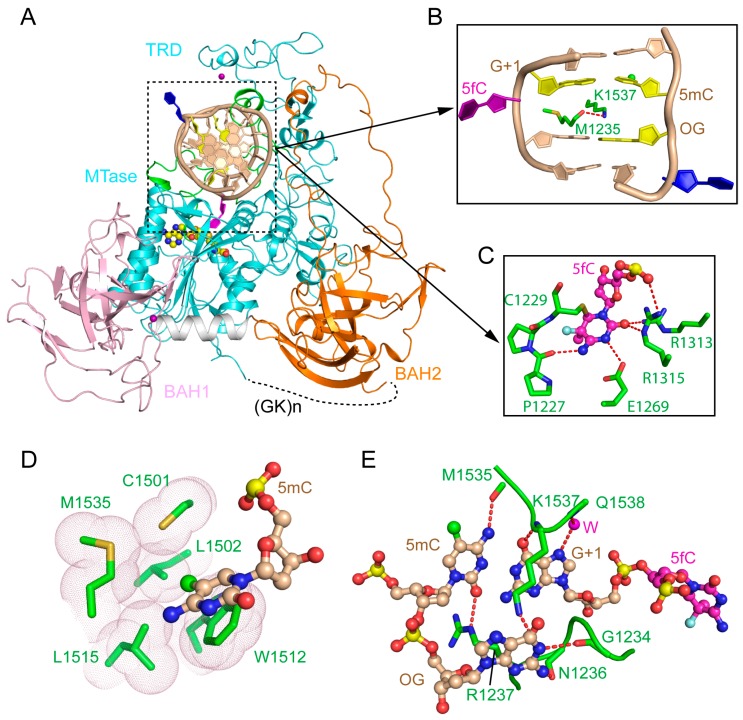
Structure of mDNMT1-DNA productive complex. (**A**) Structural overview of mDNMT1 (amino acids 731–1602) covalently bound to hemimethylated DNA (Protein Data Base (PDB) 4DA4). The zinc ions are shown in purple spheres. 5fC and another flipped-out cytosine from the template strand are colored in purple and blue, respectively. (**B**) The DNA cavity vacated by the base flipping is filled with mDNMT1 residues M1235 and K1537. (**C**) The flipped-out 5fC is surrounded by active site residues through covalent linkage or hydrogen bonding interactions. (**D**) Residues from the target recognition domain (TRD) loop II form a hydrophobic groove harboring the methyl group from 5mC. (**E**) CpG-specific interactions by the TRD loop I and the catalytic loop. 5mC: 5-methylcytosine; 5fC: 5-fluorocytosine.

The crystal structure of a mouse DNMT1 fragment (mDNMT1, residues 731–1602) covalently bound to a 12-mer hemimethylated DNA duplex provides insight into the productive state of DNMT1 (Figure 2A) [31]. The DNA molecule contains one central CpG site in which a 5-methylcytosine (5mC) and a 5-fluorocytosine (5fC) were installed on the template and target strands, respectively (Figure 2B). The use of 5fC permits the formation of an irreversible, covalent complex between mDNMT1 and DNA [34]. The mDNMT1 fragment contains the pair of BAH domains (BAH1, BAH2) and the MTase domain. 

The structure of the mDNMT1-DNA covalent complex reveals that the MTase domain, composed of a catalytic core and a large TRD (~200 amino acids), is organized into a two-lobe architecture, creating a cleft to harbor the DNA duplex (Figure 2A). The two BAH domains are separated by one α-helix, both with a tilted β-barrel fold that is reminiscent of other BAH domains (Figure 2A) [35]. Both BAH domains are structurally associated with the MTase domain, forming an integrated structural unit. The BAH1 domain is attached to the MTase domain through antiparallel β-pairing, as well as hydrophobic clustering, while the BAH2 domain interacts with the MTase domain mainly through hydrophobic contacts, with a long loop (BAH2-loop) protruding from one end of the β-barrel to join with the TRD at the tip (Figure 2A). This mDNMT1 construct also contains two Cys3His-coordinated zinc finger clusters, one located in the TRD while the other associates BAH1 with the subsequent α-helix (Figure 2A). The mDNMT1-DNA interaction spans eight base pairs, resulting in a buried surface area of ~2100 Å^2^. The target cytosine, 5fC, is flipped out of the DNA duplex and inserts into the active site of mDNMT1, where it forms a covalent linkage with the catalytic cysteine C1229, leading to hydrogen bonding interactions with a number of highly conserved residues (Figure 2C). The base flipping of 5fC creates a large cavity at the hemimethylated CpG site, which is in turn filled with bulky side chains of K1537 from the TRD and W1512 from the catalytic core (Figure 2B). This protein-DNA intercalation further shifts the orphan guanine, which is otherwise paired with the flipped-out 5fC, one base down, resulting in the flipping out of a second nucleotide from the template strand (Figure 2B). The interaction of mDNMT1 with the hemimethylated CpG site involves two loops from the TRD (TRD loop I: Residues 1501–1516 and TRD loop II: Residues 1530–1537) and one loop from the catalytic site (catalytic loop: Residues 1227–1243). Toward the DNA major groove, residues from TRD loop I form a concave hydrophobic surface to harbor the methyl group of 5mC (Figure 2D). On the other hand, residues from TRD loop II engage in base-specific hydrogen bonding interactions with the CpG site (Figure 2E). On the minor groove side, residues from the catalytic loop also form base-specific contacts with the CpG site through hydrogen bonding interactions (Figure 2E). In addition, residues from both the TRD and catalytic core are involved in salt-bridge or hydrogen-bonding interactions with the DNA backbone. The two BAH domains are positioned distant to the DNA binding site. Nevertheless, residues from the tip of the BAH2-loop contribute to the DNA binding through hydrogen bonding interactions with the DNA backbone of the target strand (Figure 2A). 

In summary, the structure of the productive mDNMT1-DNA complex provides the molecular basis for the substrate recognition of DNMT1. The extensive protein-DNA contacts underlie the processive methylation kinetics of this enzyme [36,37]. More importantly, it offers explanations on the strict substrate specificity of DNMT1 on the CpG sites, as well as on the marked substrate preference of DNMT1 toward hemimethylated CpG sites [18,28]. 

### 2.2. CXXC Domain-Mediated Autoinhibition of DNMT1

**Figure 3:**
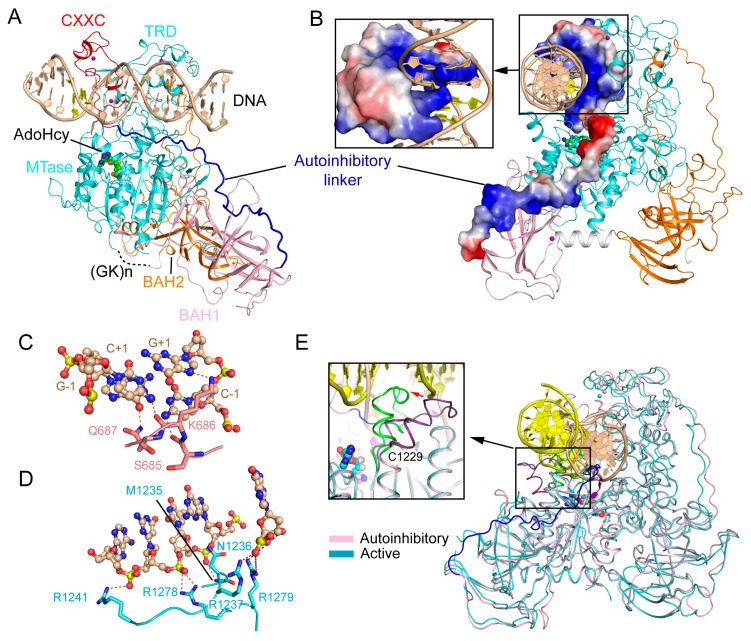
Structural analysis of the CXXC domain-mediated DNMT1 autoinhibition. (**A**) Structural overview of mDNMT1 (amino acids 650–1602) bound to a 19-mer DNA duplex containing unmethylated CpG sites (PDB 3PT6). (**B**) Surface views of the CXXC domain and the autoinhibitory linker in the complex of mDNMT1 with unmethylated CpG DNA. (**C**) Base-specific interactions between the CXXC domain and the CpG site. The hydrogen bonding interactions are depicted as dashed lines. (**D**) The MTase-DNA interactions in the autoinhibitory complex. (**E**) Structural overlay between the active (light blue) (PDB 4DA4) and autoinhibitory (pink) (PDB 3PT6) complexes of mDNMT1, with the catalytic loops highlighted in the expanded view.

The CXXC domain of DNMT1 belongs to one family of zinc finger domains that specifically bind to unmethylated CpG-containing DNA [30,38]. It manifests in a crescent-like fold, with two zinc finger clusters formed by the conserved CXXCXXC motifs in cooperation with distal cysteines. The crystal structure of an mDNMT1 fragment (residues 650–1602), spanning from the CXXC domain to the MTase domain, in complex with a 19-mer DNA duplex containing unmethylated CpG sites provides insight into the functional role of this domain (Figure 3A) [30]. In the structure, the CXXC domain is positioned on the opposite side of the MTase domain from the BAH domains, with a long CXXC-BAH1 domain linker (also known as autoinhibitory linker) running across the catalytic cleft (Figure 3A). The mDNMT1-unmethylated DNA complex contains two separate DNA-binding interfaces, one located in the CXXC domain and the other located in the MTase domain. At one end of the DNA, the CXXC domain interacts with the DNA molecule from both the major groove and the minor groove, with a loop segment (R684-S685-K686-Q687) penetrating into the CpG site for base-specific contacts (Figure 3B,C). At the other end of the DNA, the MTase domain interacts with the DNA backbone through the C-terminal portion of the catalytic loop (residues M1235, R1237 and R1241) and the adjacent α-helix (R1278 and R1279) (Figure 3D). These protein-DNA interactions together localize the DNA molecule outside the catalytic cleft, resulting in an autoinhibitory conformation of DNMT1. Structural comparison of the autoinhibitory and active states of mDNMT1 reveals that the largest conformational change of mDNMT1 lies in the catalytic loop, which is poised in a retracted conformation in the autoinhibitory state, but penetrates into the DNA minor groove in the active state (Figure 3E). Furthermore, the α-helix following the catalytic loop undergoes a kinked-to-straight conformational transition, thereby regulating the contact between the catalytic loop and the DNA minor groove (Figure 3E). Indeed, a subsequent study indicated that disruption of this conformational transition leads to the impaired enzymatic activity of DNMT1 [39], highlighting the importance of this conformational switch in DNMT1-mediated DNA methylation.

These structural observations therefore led to an autoinhibitory model of DNMT1: The CXXC domain specifically interacts with the unmethylated CpG site, which in turn stabilizes the positioning of the autoinhibitory linker over the catalytic cleft, leading to the extrusion of the unmethylated CpG DNA from the catalytic site. This model therefore assigns a regulatory role to the CXXC domain in inhibiting the de novo methylation activity of DNMT1. Indeed, enzymatic assays based on the mDNMT1(650–1602) construct indicated that disruption of the CXXC-CpG interaction or deletion of the autoinhibitory linker both led to enhanced enzymatic activity of DNMT1 on unmethylated CpG DNA, but resulted in no significant change to hemimethylated substrates, lending support to the autoinhibitory mechanism. However, it is worth noting that a later study on full-length DNMT1 failed to identify any significant impact of the CXXC-DNA interaction on the substrate specificity of DNMT1 in vitro [40], suggesting that additional factors (e.g., protein interactions or post-translational modifications) may be needed to stabilize the CXXC domain-mediated autoinhibitory conformation, thereby ensuring the substrate specificity of DNMT1 in cells.

### 2.3. RFTS Domain-Mediated Autoinhibition of DNMT1

**Figure 4:**
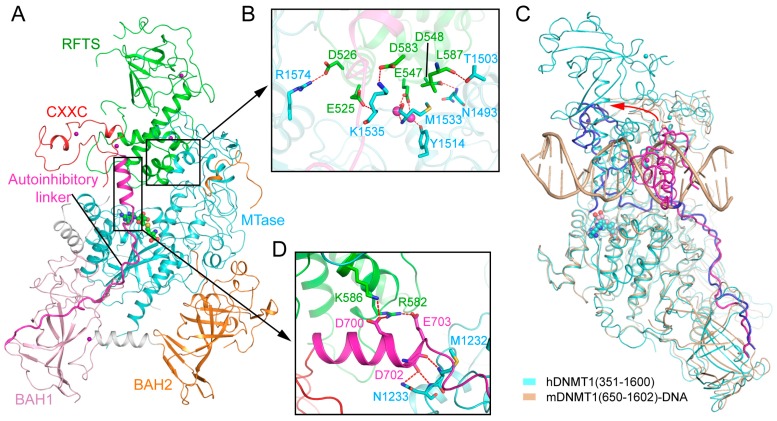
Structural analysis of the replication foci-targeting sequence (RFTS) domain-mediated DNMT1 autoinhibition. (**A**) Structural overview of hDNMT1 (amino acids 351–1602) (PDB 4WXX). (**B**) The intramolecular interactions between the RFTS (green) and MTase (aquamarine) domains. The hydrogen bonding interactions are depicted as dashed lines. The water molecules are shown as purple spheres. (**C**) Structural overlap between the CXXC (PDB 3PT6) and RFTS (PDB 4WXX) mediated autoinhibitory complexes, with the autoinhibitory linkers colored in blue and light magenta, respectively. The repositioning of the CXXC domain is indicated by a red arrow. (**D**) The interaction of the autoinhibitory linker (magenta) with both the RFTS (green) and MTase domains (aquamarine).

The crystal structures of DNA-free mouse and human DNMT1 fragments, spanning from the RFTS domain toward the MTase domain, reveal that the RFTS domain closely associates with the MTase domain, resulting in a compact fold (Figure 4A) [32,33]. In both structures, the RFTS domain folds into two lobes, separated by a 24-amino acid long α-helix (Figure 4A). The N-lobe is dominated by a zinc finger cluster, followed by a six-stranded β-barrel, while the C-lobe is assembled into a helical bundle (Figure 4A). The N and C lobes form an acidic cleft, where the linker sequence downstream of the RFTS domain extends away from the RFTS domain (Figure 4A). The intramolecular contact between the RFTS and MTase domains is underpinned by hydrogen bonding interactions between the residues from the C-lobe of the RFTS and the residues from the TRD (Figure 4B), which partially overlap with the DNA binding surface of the TRD (Figure 2A). The CXXC domain is positioned adjacent to the RFTS domain, adopting a conformation similar to its DNA-bound state (Figure 4A). Structural comparison of DNA-free DNMT1 and its unmethylated CpG DNA-bound state reveals a large conformational repositioning of the CXXC domain: It sits on one side of the TRD in the structure of mDNMT1–19-mer unmethylated CpG DNA, but moves to the front of the TRD in the structure of free DNMT1, resulting in a translocation of ~30 Å (Figure 4C). As a result, the autoinhibitory linker downstream of the CXXC domain undergoes a large conformational change between the two complexes: It runs across the catalytic cleft in the DNMT1-unmethylated CpG DNA complex but is released from the catalytic cleft in free DNMT1 (Figure 4C). Intriguingly, this repositioning of the autoinhibitory linker is accompanied by a loop-to-helix conformational transition: The N-terminal end of the linker assumes an extended conformation in unmethylated CpG-bound DNMT1 but shows a helical structure in free DNMT1 (Figure 4C). At the C-terminal end of this helix, residues D700 and E703 form salt bridges with residues R582 and K586 from the RFTS domain, while residue D702 forms hydrogen bonds with residues M1232 and N1233 from the catalytic core, which together help to strengthen the interaction between the RFTS and MTase domains (Figure 4D). Consistently, deletion of residues 701–711 from the autoinhibitory linker led to significantly enhanced enzymatic activities of DNMT1 [33]. These data therefore suggest that the autoinhibitory linker not only plays a critical role in the CXXC domain-mediated DNMT1 autoinhibition, but also contributes to the RFTS domain-mediated DNMT1 autoinhibition. 

### 2.4. Allosteric Regulation of DNMT1

**Figure 5:**
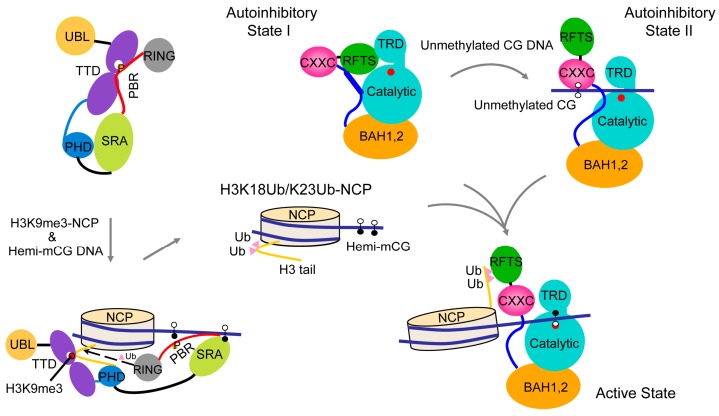
A model for the allosteric regulation of DNMT1-mediated maintenance DNA methylation. Hemimethylated DNA and histone H3K9me3 serve as epigenetic signals to promote UHRF1-mediated ubiquitination of histone H3, which in turn shifts the conformation of DNMT1 from the autoinhibitory state into an active state for maintenance DNA methylation. UHRF1 P656, which occupies the H3K9me3-binding cage of the tandem Tudor domain (TTD) in the closed UHRF1 conformation, is indicated by the letter P. The active site of DNMT1 is marked by a filled red circle.

Crystal structures of DNMT1 in a DNA-free state, in complex with unmethylated CpG DNA and in complex with hemimethylated CpG DNA together demonstrate that DNMT1 may adopt distinct conformational states under different DNA binding conditions, suggesting a multi-layered regulation of DNMT1 activity. It is conceivable that the interconversion between these states permits DNMT1 to discriminate the DNA substrates under different epigenetic environments, such as methylation-free CpG islands compared to heavily methylated heterochromatic regions (Figure 5). The stabilization of each conformation is likely to be achieved by the distinct DNA or histone-binding mode of DNMT1 under different environments, ensuring DNMT1 will replicate the DNA methylation pattern both faithfully and efficiently. Indeed, emerging studies have suggested a model in which DNMT1 mediates region-specific DNA methylation maintenance, rather than site-specific DNA methylation maintenance [41]. 

The RFTS domain mediates the localization of DNMT1 to replication foci and constitutive heterochromatin from late S throughout the G2 and M phases [42,43]. A number of mutations in the RFTS domain have been associated with neurological disorders, including hereditary sensory autonomic neuropathy with dementia and hearing loss (HSAN1E) [44,45], cerebella ataxia, deafness and narcolepsy (ADCA-DN) [46,47]. These mutations presumably affect the folding and stability of the RFTS domain [33], which in turn may lead to the dysregulation of DNMT1-mediated methylation. Recent structural and functional characterizations of the interaction between the DNMT1 RFTS domain and histone modifications have further elucidated the functional implication of the RFTS domain-mediated DNMT1 autoinhibition [48]. In particular, it has been shown that the DNMT1 RFTS domain binds to histone H3 ubiquitinated at lysine 14 (K14Ub), 18 (K18Ub) and/or 23 (K23Ub), with a preference for H3 with two mono-ubiquitination (H3Ub2) [48,49,50]. The crystal structure of the RFTS domain of hDNMT1 in complex with H3-K18Ub/K23Ub reveals that the two ubiquitin moieties engage in hydrophobic interactions with two discrete surfaces of the N-lobe of RFTS, separated by a loop segment [48]. The N-terminal tail of H3 lies between the C-lobe and the ubiquitin molecule conjugated to H3K23, leading to the eviction of the linker sequence downstream of the RFTS domain out of the cleft between the N and C lobes [48]. In accordance with these structural changes, the interaction of DNMT1 RFTS with H3Ub2 results in a substantially elevated level of activity of DNMT1 [48], suggesting that H3Ub2 may serve as an epigenetic signal that relieves the RFTS-mediated autoinhibition of DNMT1. These studies have therefore established a link between the chromatin targeting and enzymatic activation of DNMT1, unveiling the molecular mechanism for RFTS regulation (Figure 5). It is worth noting that the H3 K14Ub/K18Ub/K23Ub marks are the enzymatic products of UHRF1 (ubiquitin-like, containing plant homeodomain (PHD) and RING finger domains) [48,49,50], a key regulatory protein of DNMT1-mediated maintenance DNA methylation [51,52]. UHRF1 is also a multi-domain protein comprised of an N-terminal ubiquitin-like (UBL) domain, a tandem Tudor domain (TTD), a plant homeodomain (PHD), a SET and RING-associated (SRA) domain and a C-terminal RING finger domain [53]. An intramolecular interaction between the TTD domain and the C-terminal polybasic region (PBR) of UHRF1 results in a closed conformation that occludes UHRF1 from chromatin association [54,55,56,57]. During the S phase, the association of UHRF1 with histone H3 trimethylated at lysine 9 (H3K9me3) [58,59,60,61,62,63,64], a silencing histone mark [65], and hemimethylated CpG DNA [51,52,61,66,67,68,69] leads to the conformational opening [54,55,56], and enhanced E3 ubiquitin ligase activity of UHRF1 (Figure 5) [70]. In this context, the DNMT1 RFTS domain serves as an effector module that transmits the H3K9me3 signal into DNMT1-mediated DNA methylation (Figure 5).

### 2.5. Regulatory Role of DNMT1 N-Terminal Domain

The N-terminal domain (NTD) appears not to affect the enzymatic activity of DNMT1. Instead, this region serves as a platform for the interaction between DNMT1 and proteins or DNA. Of particular note, the fragment equivalent to residues 159–171 of mouse DNMT1 (mDNMT1) is responsible for interacting with proliferating cell nuclear antigen (PCNA) [71], thereby contributing to the recruitment of DNMT1 to the replication foci during the S phase [71], or the DNA repair sites [72]. The NTD reportedly also interacts with other proteins, including DMAP1 [73], G9a [74], DNMT3A [75], DNMT3B [75], PKC [76] and CDKL [77] to regulate transcription repression, heterochromatin formation or the pathogenic processes of Rett syndrome. In addition, the DNA binding activity of the NTD has been reported [78,79,80]. However, due to lack of a structural study, the functional implication of most of the NTD-associated interactions remains to be investigated.

### 2.6. Regulatory Role of DNMT1 (GK)n Repeats

The (GK)n repeat of DNMT1, which is highly conserved throughout evolution, links the regulatory domains to the MTase domain. Current structural studies indicate that this repeat is not involved in the DNA interaction. Rather, it constitutes a binding site for deubiquitinase USP7, an enzyme that plays a regulatory role in DNMT1-mediated maintenance DNA methylation [81,82,83,84]. The DNMT1-USP7 interaction is subject to regulation by the acetyltransferase Tip60 and the deacetylase HDAC1: Tip60-mediated acetylation of the (GK)n repeat leads to the disruption of the DNMT1-USP7 interaction, which can be restored by the HDAC1-mediated deacetylation of the same site [82]. On the other hand, a more recent study has suggested that the (GK)n repeat may participate in the DNMT1-mediated de novo methylation of paternal imprinting control regions (ICRs) in mouse ES cells [85]. Due to the lack of molecular details of DNMT1-mediated methylation in cells, the functional implication of the (GK)n repeat remains controversial [86].

## 3. Structural Basis of DNMT3A-Mediated DNA Methylation

DNMT3A and DNMT3B mediate DNA methylation establishment during gametogenesis and embryogenesis [16,87], and subsequently participate in methylation maintenance [88,89,90]. The enzymatic activity of DNMT3A/3B in germ cells and embryonic stem cells is further regulated by DNMT3-like (DNMT3L) protein, which lacks DNA methylation activity but functions to stimulate the cofactor binding and enzymatic activity of DNMT3A/3B [7,91,92,93] and to maintain DNMT3A stability in cells [94]. DNMT3A and DNMT3B are highly related in sequence, both containing a largely disordered NTD, followed by a Pro-Trp-Trp-Pro (PWWP) domain, an Atrx-Dnmt3-Dnmt3l (ADD) domain and a highly homologous MTase domain (Figure 1). DNMT3L contains an N-terminal ADD domain, followed by a MTase-like domain, which is catalytically inactive due to a lack of essential motifs for enzymatic activity (Figure 1) [95,96].

### 3.1. Enzyme-Substrate Interaction of DNMT3A

The crystal structure of the MTase domain of DNMT3A in complex with the C-terminal domain of DNMT3L (DNMT3L-C) provides the first atomic details of the DNMT3A-DNMT3L complex [97]. The DNMT3A MTase domain forms a tetrameric fold with DNMT3L-C, in the order of 3L-3A-3A-3L, resulting in two DNMT3A-DNMT3L heterodimeric interfaces and one DNMT3A-DNMT3A homodimeric interface. The homodimerization of DNMT3A is mediated by a network of salt bridges and hydrogen bonding interactions, while the heterodimerization of DNMT3A and DNMT3L is mainly driven by hydrophobic stacking interactions between two pairs of phenylalanine residues [97]. Notably, the active sites between the two DNMT3A monomers are separated by ~40 Å, a distance equivalent to one helical turn of DNA. This observation provides the basis for the CpG spacing model, in which the DNMT3A dimer is capable of methylating two CpG sites located across the opposite strands of one DNA duplex, separated by ~10 base-pair (bp) DNA, in one binding event. This model predicts the prevalence of ~10 bp methylation periodicity in cells, which has been supported by a number of biochemical and cellular studies [97,98]. However, the observation that the 10 bp-methylation periodicity also occurs in plants later prompted alternative explanations for the methylation periodicity [99].

**Figure 6:**
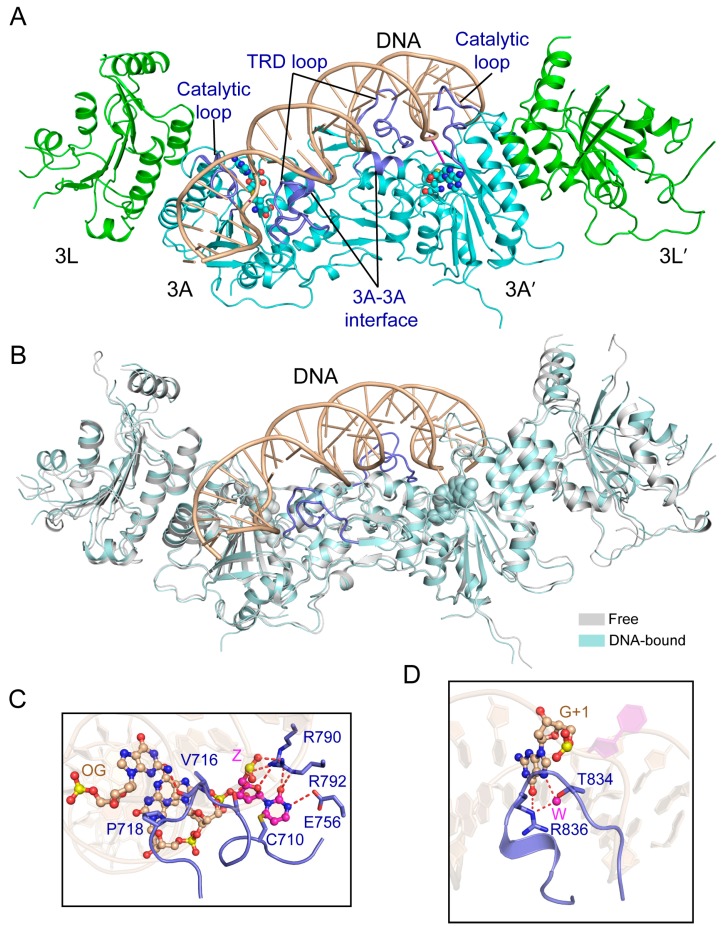
Structural analysis of the productive complex of the DNMT3A-DNMT3L tetramer with CpG DNA. (**A**) Structural overview of the DNMT3A-DNMT3L tetramer covalently bound to a 25-mer DNA duplex containing two CpG/ZpG sites (Z: Zebularine) (PDB 5YX2). The flipped-out zebularines are colored in purple. (**B**) Structural overlap between the DNA-bound and free DNMT3A-DNMT3L tetramer (PDB 2QRV). The TRD loops, which undergo disorder-to-order transition upon DNA binding, are colored in blue. (**C**) The DNA interactions involving the catalytic loop and other catalytic residues. (OG: Orphan guanine). The hydrogen bonding interactions are depicted as dashed lines. (**D**) Residues T834 and R836 from the TRD loop (blue) engage in base-specific recognition of the CpG site. The water molecules are shown as purple spheres.

Recently, the crystal structure of DNMT3A-DNMT3L in complex with a DNA duplex containing two separate CpG sites (in which the target cytosines are replaced with zebularines [100]) has been determined [101]. The structure reveals a productive state of the DNMT3A-DNA complex, with two CpG/ZpG (Z: zebularine) sites separately targeted by the two DNMT3A monomers of the DNMT3A-DNMT3L tetramer (Figure 6A), therefore confirming the notion of DNMT3A-mediated DNA co-methylation. The structure of the DNA-bound DNMT3A-DNMT3L tetramer resembles that of free DNMT3A-DNMT3L (Figure 6B), with an RMSD of 1.1 Å over 826 aligned Cα atoms. The most notable structural difference arises from a loop from the TRD (TRD loop), which undergoes a disorder-to-order transition upon DNA binding (Figure 6B). The interaction between DNMT3A and DNA is mediated through the catalytic loop, the TRD loop and the DNMT3A-DNMT3A homodimeric interface (Figure 6A), which together create a continuous DNA-binding surface. The zebularines are flipped out of the DNA duplex and insert deep into the catalytic pocket of DNMT3A, where they are covalently anchored by the catalytic cysteine C710 and recognized by several other residues through hydrogen bonding interactions (Figure 6C) [101]. Similar to the productive mDNMT1-DNA complex, the catalytic loop and TRD loop of DNMT3A approach the DNA molecule from the minor groove and the major groove, respectively, with residue V716 from the catalytic loop intercalating into the DNA cavity vacated by base flipping (Figure 6C,D). In the minor groove, the backbone carbonyl of V716 forms a hydrogen bond with the orphan guanine (Figure 6C), while in the major groove, residues R836 and T834 from the TRD loop also interact with the guanine of the target strand through direct and water-mediated hydrogen bonding interactions (Figure 6D). Consistent with these structural observations, the introduction of mutations into these CpG-interacting residues leads to either dramatically decreased activity (for V716G) or altered methylation specificity (for R836A) in vitro and in cells [101]. Mutations of the substrate binding site of DNMT3A, including R882H, have been associated with hematological cancer [102,103,104]. Both in vitro and in vivo assays indicated that these mutations compromise the enzymatic activity of DNMT3A [101,102,103,104,105,106,107], which may contribute to disease progression.

It is worth noting that the structure of the DNMT3A-DNMT3L- DNA complex reveals that the active sites between the two DNMT3A monomers are separated by 14 bp DNA, instead of the 10 bp as previously proposed. Whether this observation arises from the inherent structural property of DNMT3A or its conformational dynamics remains to be investigated.

### 3.2. ADD Domain-Mediated Autoinhibition of DNMT3A

The ADD domain of DNMT3A is comprised of an N-terminal GATA-like zinc finger, a PHD finger and a C-terminal α-helix [108], together packing into a single globular fold. This domain has been characterized as a reader module that specifically binds to histone H3 unmethylated at lysine 4 (H3K4me0) [108,109]. The association of the DNMT3A ADD domain with H3K4me0 is mediated by antiparallel β-pairing between the two-stranded β-sheet of the ADD domain and residues A1-T6 of H3, with the side chain of H3K4me0 engaging in hydrogen-bonding interactions with D529, D531 and Q534 from the ADD domain [108]. In addition, a downstream loop of the ADD domain undergoes a disorder-to-order transition to close up on the N-terminus of H3, supporting the specific ADD-H3 association [108]. 

**Figure 7:**
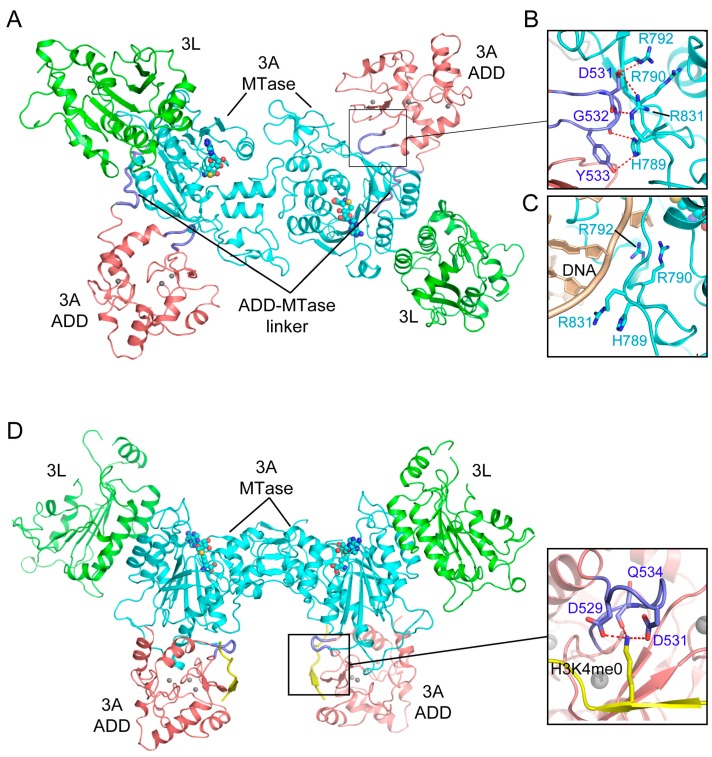
Structural analysis of the Atrx-Dnmt3-Dnmt3l (ADD) domain-mediated DNMT3A autoinhibition. (**A**) Structural overview of the DNMT3A-DNMT3L tetramer, with the DNMT3A fragment comprised of both the ADD and MTase domains (PDB 4U7P). (**B**) Intramolecular interactions between the ADD loop (blue) and the MTase domain (aquamarine) of DNMT3A. The hydrogen bonding interactions are depicted as dashed lines. (**C**) The ADD-binding site of the DNMT3A MTase overlaps with its DNA binding site. (**D**) Structure of the DNMT3A-DNMT3L tetramer bound to the histone H3K4me0 peptide (PDB 4U7T), with the interaction between H3K4me0 and the ADD domain shown in an expanded view (PDB 3A1B).

Recent studies have further revealed that the ADD domain regulates the activity of DNMT3A through an H3-dependent, autoinhibitory mechanism [110,111]. The structure of a DNMT3A fragment, spanning the ADD and MTase domains, in complex with DNMT3L-C reveals an intramolecular interaction between the ADD and MTase domains of DNMT3A (Figure 7A). In particular, the linker sequence following the ADD domain initiates a hydrophobic contact with the MTase domain, which then guides the insertion of a loop (residues 526–533) of the ADD domain into the catalytic cleft, where it engages in salt-bridge interactions with DNA binding sites (R790, R792, H789 and R831) (Figure 7B), thereby inhibiting the substrate binding of DNMT3A (Figure 7C) [110]. In contrast, the structure of the DNMT3A-DNMT3L-H3 complex demonstrates that, upon binding to H3 (Figure 7D,E), the DNMT3A ADD domain is repositioned from the catalytic cleft onto a different surface of the MTase domain, engaging a distinct set of hydrogen bonds and hydrophobic interactions (Figure 7D) [110]. The structural comparison of the H3-free and H3-bound DNMT3A complexes therefore provides a dynamic view on how the H3 binding switches the conformation of DNMT3A from an autoinhibitory state to an active state. Note that the residues involved in the autoinhibitory regulation of DNMT3A are highly conserved in DNMT3B, suggesting a conserved allosteric regulation mode of DNMT3 methyltransferases. 

The observation that the intramolecular ADD-MTase interaction interplays with the intermolecular ADD-H3 interaction establishes a direct coupling between the enzymatic activity and chromatin targeting of DNMT3A. Similar to the RFTS domain-mediated allosteric regulation of DNMT1, as described above, this regulatory mechanism of the DNMT3A ADD domain ensures the precise spatial regulation of DNMT3A [109,110,111], which is essential for installing lineage-specific DNA methylation patterns across the genome.

### 3.3. Functional Regulation of DNMT3A by the N-Terminal Tail and PWWP Domain

The NTD segment defines the most divergent region between DNMT3A and DNMT3B. This region has been shown to regulate the DNA binding and cellular localization of DNMT3A [112,113,114]. Unlike full-length DNMT3A that is predominantly localized to the heterochromatic region, DNMT3A2, an isoform of DNMT3A lacking residues 1–221 of the NTD, becomes enriched in the euchromatic region, with reduced DNA binding affinity [114]. The precise regulatory role of this domain remains to be investigated. 

**Figure 8:**
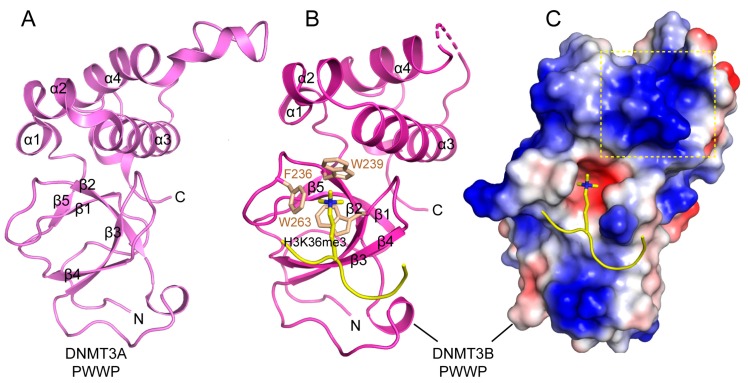
Structures of DNMT3A/3B Pro-Trp-Trp-Pro (PWWP) domains. (**A**) Crystal structure of the DNMT3A PWWP domain (PDB 3LLR). (**B**) Crystal structure of DNMT3B PWWP bound to the histone H3K36me3 peptide (PDB 5CIU), with the side chains of H3K36me3 and the cage residues of the PWWP domain shown in stick representation. (**C**) Electrostatic surface view of the DNMT3B PWWP domain bound to the histone H3K36me3 peptide. The putative DNA binding surface is boxed with dotted lines.

The PWWP domain, named after a characteristic proline-tryptophan-tryptophan-proline motif, belongs to the Royal super-family of domains that recognize histone tails with various modifications [115,116]. The PWWP domain of DNMT3A and DNMT3B mediates their chromatin association through specific recognition of histone H3 trimethylated at lysine 36 (H3K36me3) [117,118], which is essential for directing the de novo methylation activity of DNMT3A/3B at the pericentric heterochromatin [119]. Structural studies of the DNMT3A/3B PWWP domain revealed a β-barrel followed by a C-terminal helical bundle, similar to other PWWP domains (Figure 8A,B) [120,121,122]. The β-barrel is comprised of five β-strands, with the signature PWWP motif replaced by a SWWP motif at the beginning of the second β-strand. The structure of the DNMT3B PWWP domain in complex with an H3K36me3 peptide reveals that the histone peptide occupies a surface groove formed by residues from the β1 strand, the β1-β2 loop, and the β4 strand, with the side chain of H3K36me3 inserting into the aromatic cage formed by F236, W239 and W263 through hydrophobic and cation-π interactions (Figure 8B) [121]. The H3K36me3 binding also induces a conformational change of the β1-β2 loop, which moves to close up the aromatic cage, thereby enhancing the specific H3K36me3 recognition. In addition, both the DNMT3A and DNMT3B PWWP domains present a positively charged surface that confers their DNA binding activity (Figure 8C) [120,121,123]. The cooperative engagement of both DNA and H3K36me3 by the DNMT3A/3B PWWP domains provides a mechanism for targeting these two enzymes to heterochromatic regions [118,119] or the actively transcribed gene body in the nucleus [124]. 

## 4. Structural Comparison of the DNMT1-DNA and DNMT3A-DNA Complexes

**Figure 9:**
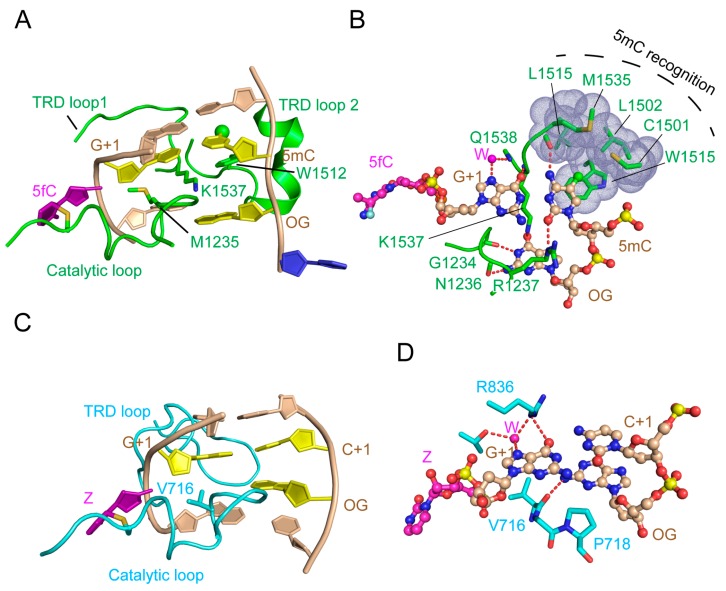
Structural comparison of mDNMT1-DNA and DNMT3A-DNA interactions. (**A**) Recognition of hemimethylated CpG DNA by mDNMT1 (green) (PDB 4DA4). The hemimethylated CpG site, containing a 5-methyl group (green sphere), is colored in yellow or purple (5-fluorocytosine, 5fC′). The flipped-out cytosine on the template strand is colored in blue and the rest of the DNA is colored in wheat. (**B**) Detailed interactions between mDNMT1 and the hemimethylated CpG site. (C) Recognition of unmodified CpG DNA by DNMT3A (light blue). (**D**) Detailed interactions between DNMT3A and the unmodified CpG site. The hydrogen bonds are depicted as dashed lines.

The structural comparison of the DNMT3A-DNMT3L-DNA complex and the mDNMT1-hemimethylated DNA complex provides insights into the distinct molecular basis between DNMT3A-mediated de novo DNA methylation and DNMT1-mediated maintenance DNA methylation. Despite the conformational similarity in their catalytic loop for accessing the DNA minor groove, mDNMT1 and DNMT3A enter the DNA major groove differently for CpG recognition (Figure 9A–D). Firstly, mDNMT1 interacts with the DNA major groove through two of its TRD loops, with one (TRD loop 1) engaging the CpG dinucleotide through hydrogen bonding interactions and the other (TRD loop 2) forming a hydrophobic concave harboring the methyl group of 5mC along the template strand (Figure 9A,B). In contrast, while DNMT3A interacts with the DNA major groove through a loop similar to TRD loop 1 in DNMT1, it lacks the DNMT1 TRD loop 2-equivalent segment for 5mC recognition (Figure 9C,D). These observations explain why DNMT1, but not DNMT3A, shows an enzymatic preference for hemimethylated substrates over unmethylated substrates. Additionally, the DNA molecules bound to mDNMT1 and DNMT3A also exhibit different conformational adjustments. In mDNMT1-bound DNA, the base flipping leads to one-base translocation of the orphan guanine and a large distortion of the CpG site, with the DNA cavity filled by two bulky protein residues (M1235 and K1537) (Figure 9A). In contrast, in DNMT3A-bound DNA, the orphan guanine remains in space, resulting in a smaller DNA cavity occupied by one small residue of DNMT3A (V716) (Figure 9C). In addition, the large TRD of DNMT1 permits an extensive protein-DNA interaction, resulting in a buried surface area of ~2100 Å^2^, whereas the DNA binding of DNMT3A, with a much smaller TRD, only leads to buried surface area of ~1300 Å^2^ for each DNMT3A monomer. This limited DNA binding of each DNMT3A monomer is nevertheless overcome by the presence of two DNMT3A monomers in the DNMT3A-DNMT3L tetramer, which provides an enlarged protein-DNA contact surface to ensure the efficiency of DNA methylation. Together, these observations highlight the molecular basis underlying the difference between DNMT3A-mediated de novo methylation and DNMT1-mediated maintenance methylation.

## 5. Summary

Recent structural and biochemical studies have greatly advanced our understanding of DNMT1-mediated maintenance DNA methylation and DNMT3A/3B-mediated de novo DNA methylation. Structural elucidations of DNMT1 and DNMT3A in complex with their respective DNA substrates or histone peptides provide mechanistic details for the functional regulation and substrate specificity of these enzymes. However, a number of outstanding questions remain to be addressed, for example, how are the N-terminal domains of DNMT1 or DNMT3A coordinated in regulating the enzymatic activity and genome targeting? How are the DNMTs regulated in the chromatin environment? Future investigations of the structure and dynamics of DNMT1 and DNMT3A/3B in their cellular environment will help provide a systematic view on the mechanistic basis of mammalian DNA methylation.
